# Comparative genomics and proteomics analysis of phages infecting multi-drug resistant *Escherichia coli* O177 isolated from cattle faeces

**DOI:** 10.1038/s41598-023-48788-w

**Published:** 2023-12-05

**Authors:** Peter Kotsoana Montso, Andrew M. Kropinski, Fortunate Mokoena, Rian Ewald Pierneef, Victor Mlambo, Collins Njie Ateba

**Affiliations:** 1 Food Security and Safety Focus Area, Faculty of Natural and Agricultural Sciences, North-West University, Private Bag X2046, Mmabatho, 2735 South Africa; 2 Department of Microbiology, Faculty of Natural and Agricultural Sciences, North-West University, Private Bag X2046, Mmabatho, 2735 South Africa; 3 Department Food Science, and Pathobiology, Ontario Veterinary College, University of Guelph, Guelph, ON N1G 2W1 Canada; 4 Department of Biochemistry, Faculty of Natural and Agricultural Sciences, North-West University, Mmabatho, South Africa; 5 Department of Biochemistry, Genetics and Microbiology, University of Pretoria, Pretoria, 0001 South Africa; 6 Centre for Bioinformatics and Computational Biology, University of Pretoria, Pretoria, 0001 South Africa; 7 SARChI Chair: Marine Microbiomics, Microbiome@UP, Department of Biochemistry, Genetics and Microbiology, University of Pretoria (UP), Hatfield, Pretoria, South Africa; 8 Faculty of Agriculture and Natural Sciences, School of Agricultural Sciences, University of Mpumalanga, Mbombela, 1200 South Africa

**Keywords:** Computational biology and bioinformatics, Molecular biology

## Abstract

The increasing prevalence of antimicrobial-resistant (AMR) pathogens has become a major global health concern. To address this challenge, innovative strategies such as bacteriophage therapy must be optimised. Genomic characterisation is a crucial step in identifying suitable phage candidates for combating AMR pathogens. The aim of this study was to characterise seven phages that infect the *Escherichia coli* O177 strain using a whole genome sequencing. The analysis of genome sequences revealed that these phages had linear dsDNA, with genome sizes spanning from 136, 483 to 166,791 bp and GC content varying from 35.39 to 43.63%. Taxonomically, the phages were classified under three different subfamilies (*Stephanstirmvirinae*, *Tevenvirinae*, and *Vequintavirinae*) and three genera (*Phapecoctavirus*, *Tequatrovirus*, and *Vequintavirus*) within the class *Caudoviricetes*. In silico PhageAI analysis predicted that all the phages were virulent, with confidence levels between 96.07 and 97.26%. The phage genomes contained between 66 and 82 ORFs, which encode hypothetical and putative functional proteins. In addition, the phage genomes contained core genes associated with molecular processes such as DNA replication, transcription modulation, nucleotide metabolism, phage structure (capsid and tail), and lysis. None of the genomes carried genes associated with undesirable traits such as integrase, antimicrobial resistance, virulence, and toxins. The study revealed high genome and proteome homology among *E. coli* O177 phages and other known *Escherichia* phages. The results suggest that the seven phages are new members of the genera *Phapecoctavirus*, *Tequatrovirus*, and *Vequintavirus* under the subfamilies *Stephanstirmvirinae, Tevenvirinae,* and *Vequintavirinae*, respectively.

## Introduction

Bacteriophages (phages), the viruses that infect and kill their bacterial host, are most abundant biological entity in the biosphere^1,2^. Phages are classified into temperate and lytic groups based on their reproductive strategies and how these affect their bacterial host^3^. The ability of lytic phages to lyse bacterial cell hosts has attracted the interest of pharmacologists and researchers in search of alternative antimicrobials . According to the International Committee of Taxonomy of Viruses (ICTV) and Bacterial and Archaeal Viruses Subcommittee, the most common lytic phages are linear double-stranded DNA (dsDNA) and tailed, belonging to the class *Caudoviricetes*^4^. The *Caudoviricetes* encompass several subfamilies including *Stephanstirmvirinae*, *Tequatrovirinae*, and *Vequintavirinae*^4^. Whole genome sequencing shows that phages have diverse genome sizes and levels of organisation^5,6^. Phages with genome sizes less than 200 kbp are classified as small or medium phages while those with genome sizes greater than 200 kbp but less than 500 kbp are referred to as jumbo phages^5,7,8^. There are over 22,000 complete phages in the NCBI database, with 21 419 classified as small or medium phages^9^. Most of the viruses with small or medium genomes belong to the *Caudoviricetes* class^7^.

Because of their diverse genome architecture, phages may infect the same and/or different bacteria species such as *Acinetobacter baumannii*, *E. coli*, *Klebsiella pneumoniae*, *Pseudomonas aeruginosa*, and *Vibrio alginolyticus* species^10–14^. Despite this, phages that infect the same host may differ significantly in terms of their genome sequences^6^. Several studies have also reported that phage genomes contain a plethora of unique genes encoding hypothetical and putative functional proteins^5,13,14^. In addition, lytic phages possess genes responsible for genome replication and nucleotide metabolism, as well as DNA and RNA polymerases, which regulate gene expression^5,15^. Other small phages carry specialised genes encoding tRNAs and aminoacyl-tRNA synthetase^10,15–17^. Phage genomes may also carry anti-CRISPR (Acr) proteins, which interact with host CRISPR-Cas system^18^. In addition, lytic phages possess genes encoding proteins responsible for the formation of a phage pseudo-nucleus, which may provide phage protection against bacterial defence mechanisms such as clustered regularly interspaced short palindromic repeats (CRISPR-Cas) system and/or restriction modified enzymes^19^. The concerted action of these features does not only make lytic phages less dependent on the host replication machinery, but also enhance phage virulence and host range^5,7^. It is these attributes that make lytic phages ideal for biocontrol application^7^. Given that lytic phages harbour an array of genes with unknown function, there is a need to determine genetic diversity and understand the evolutionary strategies that they use to overcome host defence mechanisms. Therefore, to expand on our previous study^20^, the current study determines genomic and proteomic characteristics of phages infecting *E. coli* O177 strain.

## Results

A summary of the genomic features of the seven phage genomes characterised in this study is shown in Table 1. All *E. coli* O177 phages were linear double-stranded DNA (dsDNA), varying in size from 136, 483 to 166,791 bp with a GC content between 35.39 and 43.63% (Table 1). Based on the BLASTn and PhageAI analysis, phages belonged to the class *Caudoviricetes*, under three different subfamilies (*Stephanstirmvirinae*, *Tevenvirinae*, and *Vequintavirinae*) and three genera (*Phapecoctavirus*, *Tequatrovirus*, and *Vequintavirus*) (Table 1). In silico PhageAI analysis predicted all the phages as virulent, with confidence levels between 96.07 and 97.26%. These high confidence levels suggest that these phages are likely to be lytic in nature. BLASTn analysis revealed that the *E. coli* O177 phage genomes had high sequence similarity (> 95%) to other *Escherichia* phages genomes from the NCBI database (Table S1). The total number of coding sequences (CDS) identified in phage genomes ranged from 220 to 284. Those CDS/genes encode structural proteins (major capsid, baseplate, and tail fiber), host lysis (endolysin and lysozyme), and other functions (DNA replication/transcription, repair/packaging proteins). No genes encoding undesirable (antimicrobial resistance, virulence, toxins, mobile genetic material determinants) features or anti-CRISPR proteins were detected in all the phage genomes.

**Table 1:** Genomic features of *E. coli* O177 phages.

Features	vB_EcoM_3A1_SA_NWU	vB_EcoM_10C2_SA_NWU	vB_EcoM_10C3_SA_NWU	vB_EcoM_11B_SA_NWU	vB_EcoM_12A_SA_NWU	vB_EcoM_118_SA_NWU	vB_EcoM_366V_SA_NWU
GenBank accession numbers	OR062524	OR062525	OR062526	OR062527	OR062528	OR062529	OR062530
Taxonomic features							
Class	*Caudoviricetes*	*Caudoviricetes*	*Caudoviricetes*	*Caudoviricetes*	*Caudoviricetes*	*Caudoviricetes*	*Caudoviricetes*
Subfamily	*Stephanstirmvirinae*	*Vequintavirinae*	*Stephanstirmvirinae*	*Tevenvirinae*	*Vequintavirinae*	*Stephanstirmvirinae*	*Vequintavirinae*
Genus (based on BLASTn and PhageAI)	*Phapecoctavirus*	*Vequintavirus*	*Phapecoctavirus*	*Tequatrovirus*	*Vequintavirus*	*Phapecoctavirus*	*Vequintavirus*
Lifestyle (based on PhageAI)	Virulent (confidence = 96.07%)	Virulent (confidence = 97.06%)	Virulent (confidence = 96.47%)	Virulent (confidence = 96.43%)	Virulent (confidence = 97.01%)	Virulent (confidence = 96.48%)	Virulent (confidence = 97.26%)
Genome features							
Nucleic acid	dsDNA	dsDNA	dsDNA	dsDNA	dsDNA	dsDNA	dsDNA
Genome size (bp)	150, 431	136, 476	151,980	166,791	136,483	151,980	136,485
GC content (%)	39.06	43.63	39.10	35.39	43.63	39.11	43.63
Number of features	282	220	284	277	222	284	223
Number of genes predicted	271	215	273	266	217	273	218
tRNAs	11	5	11	11	5	11	5

**Figure 1:**
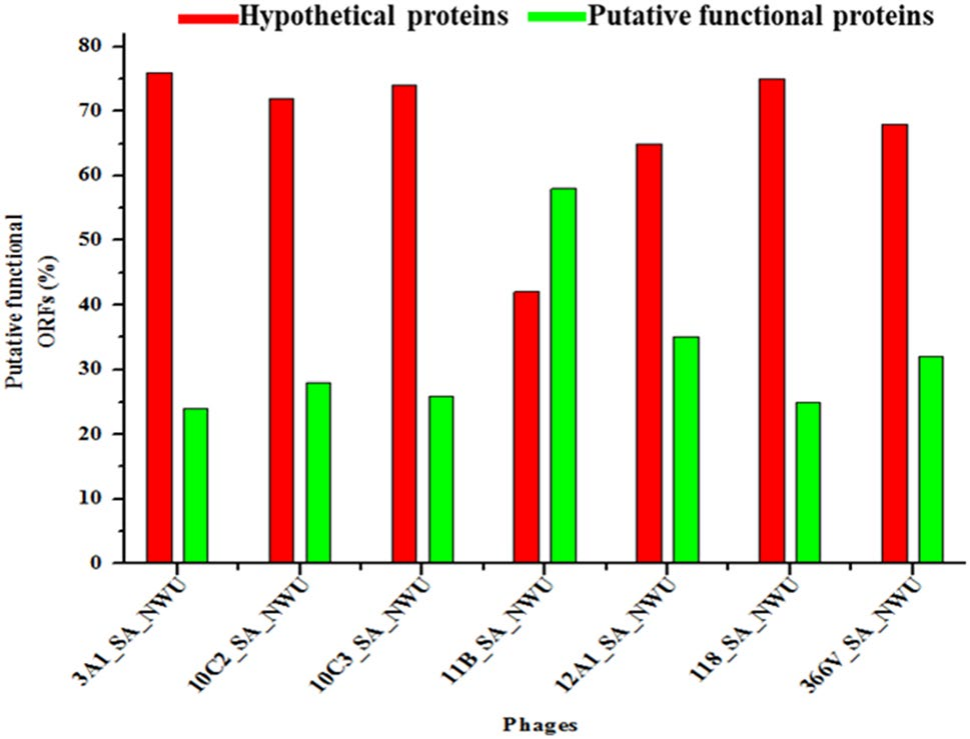
Bar graph showing the percentage of ORFs assigned to putative functional proteins in *E. coli* O177 phage genomes.

Phage genomes encompassed between 66 and 82 ORFs, with 42 to 76% predicted as hypothetical proteins and 24 to 58% assigned to various putative functional proteins. The ORFs assigned to putative functional proteins were classified into four distinct modules, namely, DNA replication and regulation, DNA packaging, structural modules (major capsid and tail), and host lysis. As depicted in Fig. 1, 58% of ORFs encoding putative functional proteins were found in phagevB_EcoM_11B_SA_NWU. The tRNAscan-SE analysis showed that all the phage genomes contained tRNA features (Table 1 and Table S2A–G). The phage genomes harboured rho–independent transcription terminators sites (ranging from 37 to 48 sites) with stem-loop secondary structure (ΔG ≤ 9 kcal mol^−1^), (Table S3A–G).

**Figure 2:**
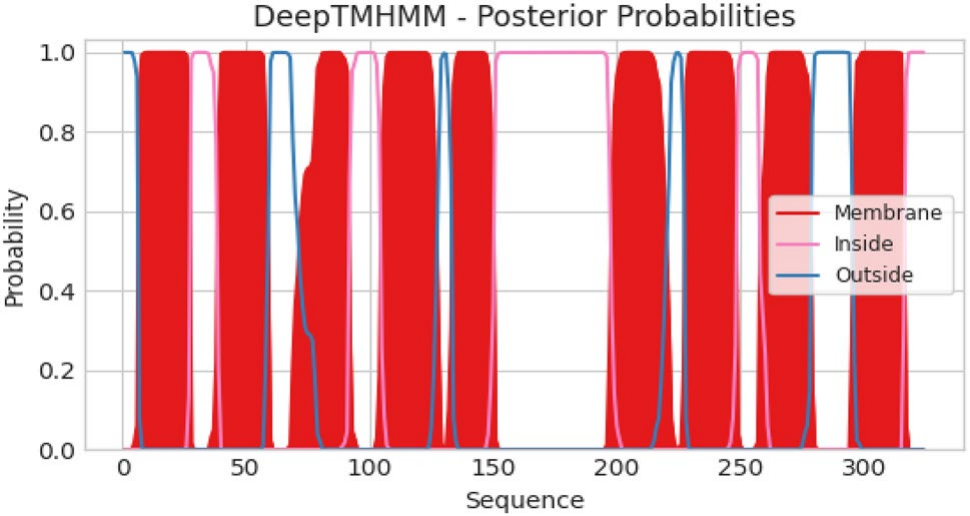
Predicted transmembrane topology of integral membrane protein using DEEPTMHMM server. The red colour shows transmembrane domains, sequence position and the ordinates represent the predicted probability.

**Figure 3:**
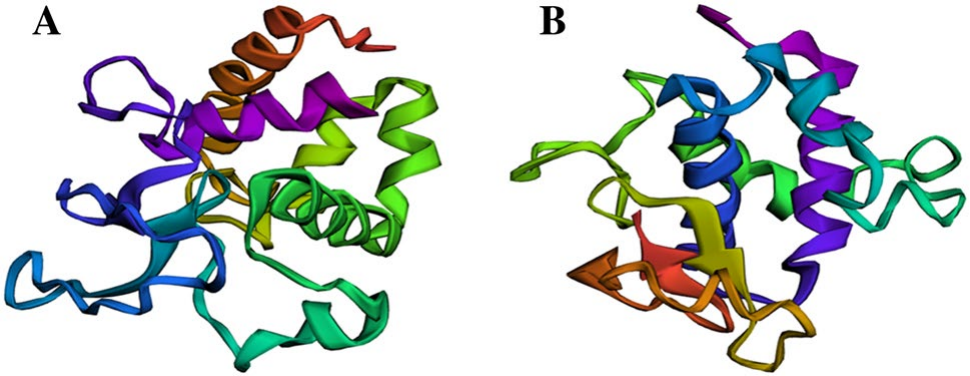
The representative 3D structure models of lysozyme (**A**), and endolysin (**B**) proteins found in *E. coli* O177 phage genomes generated using MODELLER (v10.0) with low refinement. The rainbow colour from blue to red represents N-terminus and C-terminus of the polypeptide chains, respectively.

Topology analysis showed that integral membrane protein found in phages vB_EcoM_3A1_SA_NWU, vB_EcoM_10C3_SA_NWU, and vB_EcoM_118_SANWU possessed 9 transmembrane domains (Fig. 2). However, no signal peptides were detected in the phage proteins. Table 2 indicates the physiochemical properties and secondary structure of lysin, and endolysin proteins. ExPASY ProtParam analysis indicated that lysin, and endolysin are large proteins with molecular weight of 17,423.00 and 19,779.16 kDa and Isoelectric point of 9.13 and 9.98, respectively. Both proteins were classified as stable, with the instability index (II) of 37.11 (lysozyme) and 27.11 (endolysin). Lysozyme and endolysin proteins from this study were found to be closely related (sharing ≥ 99% amino acids sequence identity) to 1AM7_A lysozyme and 5B2G_C endolysin proteins found in Enterobacteria phage lambda and Enterobacteria phage T4, respectively. MODELLER (v10.0) program was used to generate homology model of these three proteins. Figure 3 depicts 3D model of lysozyme, and endolysin containing α-helices (> 40%) random coils (> 35%), and lower frequencies of β-sheets. VERIFY_3D-1D and PROCHECK revealed reliability and quality (with > 80% of the residues averaged 3D-1D score greater than 0.2) of the predicted models. Ramachandran plot revealed that ≥ 88.1% residues of the models were placed within the favourable regions while ≤ 11.5% were within the additionally allowed region (Fgure S1). No residues were observed in the disallowed regions. PROSA Z-score of the lysozyme and endolysin were − 6.14 and − 4.08, respectively, which indicate that predicted models fall within a reasonable range of NMR and X-ray structures (Figure S2, 3). The global model quality estimate (GMQE) and Qmean values ranged from 0.6 to 0.91 and 0.46 ± 0. 06 to 0.82 ± 0.07, respectively. Overall, all the models were of high quality.

**Table 2:** Parameters and secondary structures of lysozyme and endolysin computed using Expasy ProtParam and SOPMA tools, respectively.

Characteristics	Proteins	
	Lysozyme	Endolysin
Physiochemical properties		
Number of amino acid	156	173
Molecular weight (g/mol)	17,423	19,779
pI	9.13	9.98
II	37.11	27.11
AI	83.21	87.34
Gravity	− 0.499	− 0.261
Estimated life span (hrs)	30	30
In vitro		
Mammalian reticulocytes	≥ 20	≥ 20
In vivo		
Yeast	≥ 10	≥ 10
*E. coli*		
Secondary structure		
Number of amino acid	156	173
α-Helix (Hh) %	45.81	41.04
β-turn (Tt) %	7.74	9.25
Extended strand (Ee) (%)	7.74	11.56
Random coils (%)	38.71	38.15

**Figure 4:**
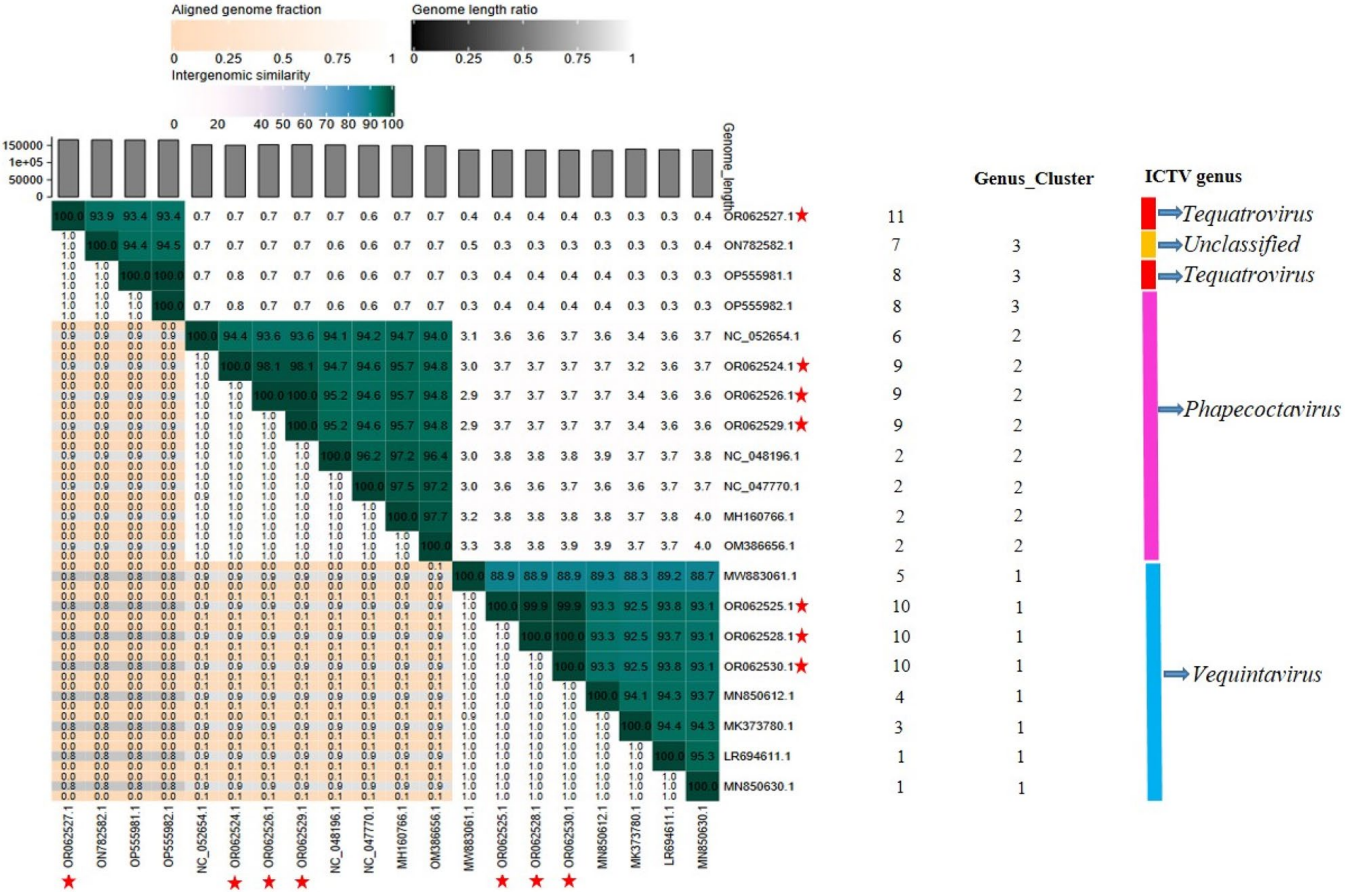
The heatmap showing percentage intergenomic sequence similarities (upper right half) and alignment genome fraction and genome length ratios (lower left half) for *E. coli* O177 phages and their closely related four *Escherichia* phages computed using VIRIDIC. The horizontal and vertical coordinates depict the corresponding phages GenBank accession number and the *E. coli* O177 phages in this study are indicated by the red asterisk (*) symbol next to their accession numbers accession number.

**Figure 5:**
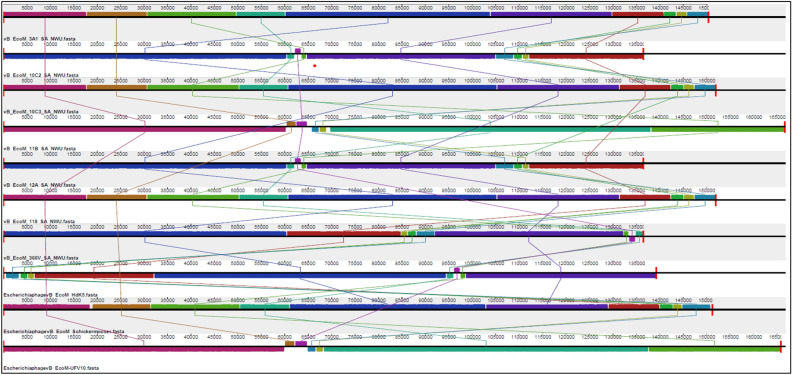
ProgressiveMauve alignment of the complete genomes of ten *Escherichia* phages [seven *E. coli* O177 phages and three reference phages (*Escherichia* phage vB_EcoM Hdk5 (accession no: MK373780.1), *Escherichia* phage vB_EcoM_Schickermooser (accession no: NC_048196.1), and *Escherichia* phage vB_EcoM UFV10 (accession no. OP555981.1)] representing three genera (*Phapecoctavirus*, *Tequatrovirus*, and *Vequintavirus*). Genome similarity is indicated by a similarity plot within the coloured blocks with the height of the plot proportional to the average nucleotide identity. The fragments that are not aligned or specific to a particular genome are represented by white areas. The regions of homologous DNA shared among the genomes are defined as local collinear blocks (LCBs) presented by boxes with the identical colours. LCBs above the genome’s center line are in the forward orientation relative to the reference genome while LCBs below the genome’s center line are in the reverse complement orientation relative to the reference genome.

**Figure 6:**
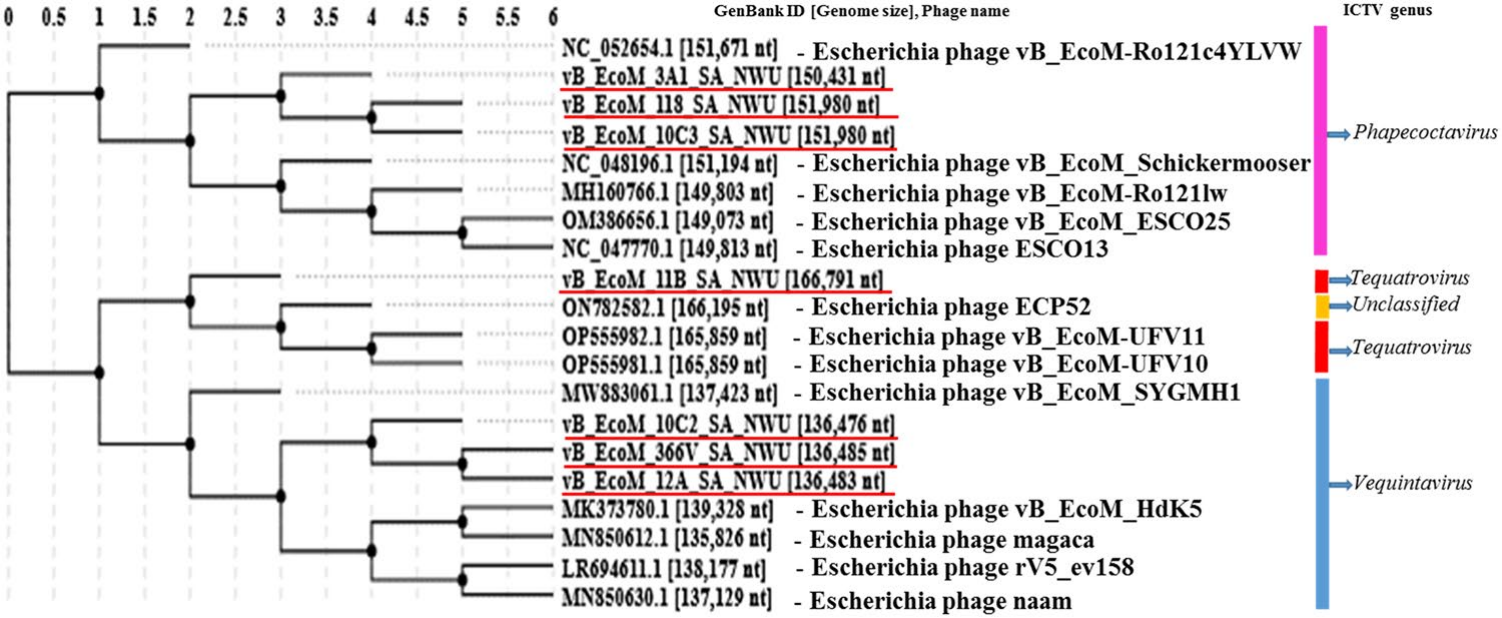
Whole genome phylogenetic tree of seven *E. coli* O177 phages with 13 selected closely related phages from the NCBI database. *E. coli* O177 phages in this study are underlined in red.

**Figure 7:**
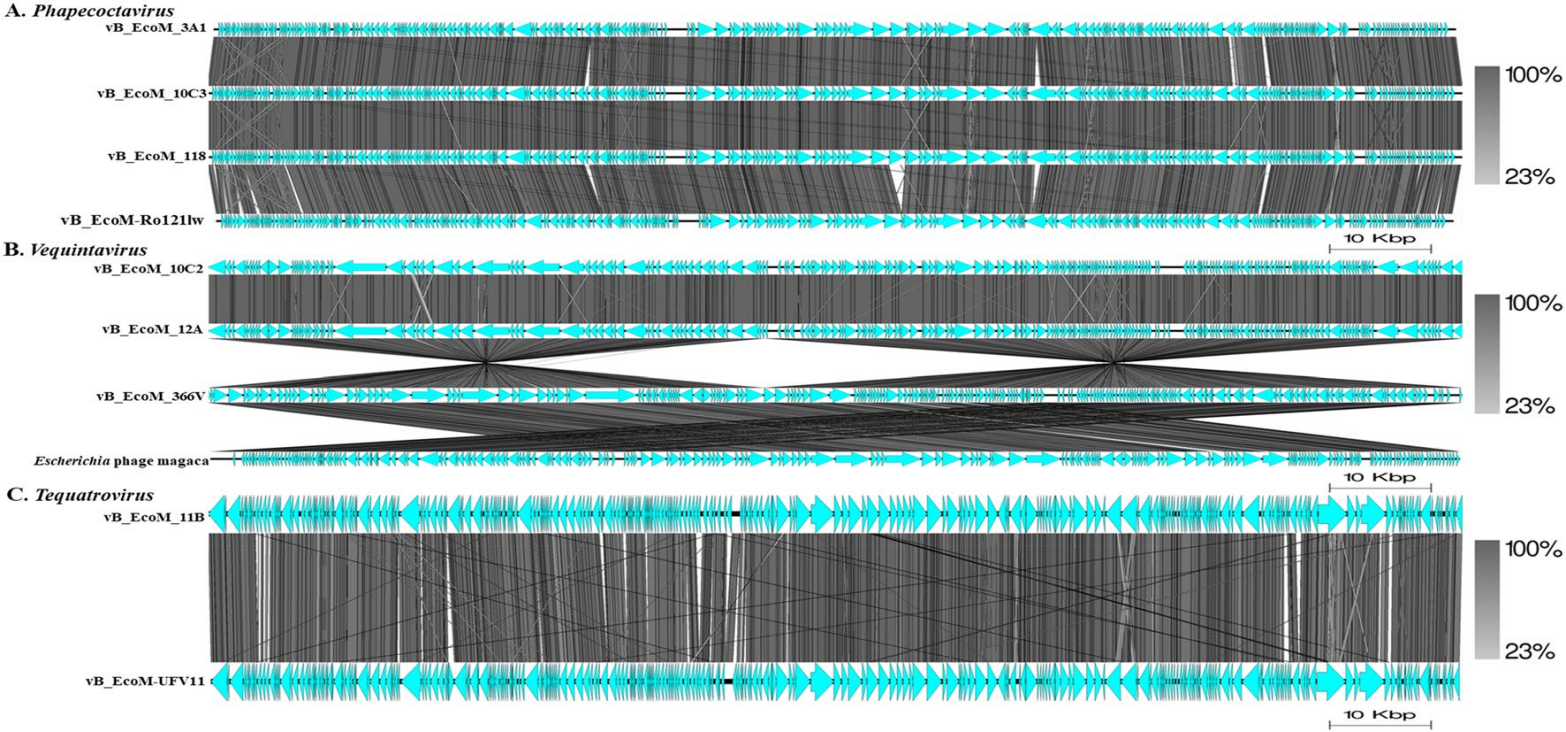
Comparison of the genome sequences of *E. coli* O177 phages with closely related members of *Phapecoctavirus* (**A**), *Vequintavirus* (**B**), and *Tequatrovirus* (**C**) genera, created using Easyfig. The grey colour between the genome maps indicates level of homology with the scales representing the percentage genome identity between the regions obtained through tBLASTx. The arrows represent the genes/CDSs. Genomes are drawn to scale; the scale bar indicates 10 Kbp.

The VIRIDIC analysis (which uses a similar algorithm to that used by the International Committee on Taxonomy of Viruses (ICTV) and Bacterial and Archaeal Viruses Subcommittee) showed high (≥ 93.7%) intergenomic similarity between *E. coli* O177 phages and related *Escherichia* phages (Fig. 4). The intergenomic similarity of three phages from the current study (vB_EcoM_3A1_SA_NWU, vB_EcoM_10C3_SA_NWU, and vB_EcoM_118_SA_NWU) and *Escherichia* phage vB_EcoM-Ro121lw (accession no. MH160766.1) was 95.7%, indicating that these phages belong to the same species. VIRIDIC analysis also showed that four phages from the current study (vB_EcoM_10C2_SA_NWU, vB_EcoM_11B_SA_NWU, vB_EcoM_12A_SA_NWU, and vB_EcoM_366V_SA_NWU) had 93.7 to 93.9% intergenomic similarity to *Escherichia* phage ECP52 (accession no. ON782582.1) and *Escherichia* phage Rv5_ev158 (accession no. LR694611.1), suggesting that they belong to the same genus (default VIRIDIC similarity threshold of 70% for genus and 95% for species). Based on progressiveMauve alignment analysis, phages within the genera *Phapecoctavirus* and *Vequintavirus* contain ten locally collinear blocks (LCBs) while phages from *Tequatrovirus* had seven LCBs (Fig. 5). *Escherichia coli* O177 phages showed similar LCBs with closely related *Escherichia* phages. The phages within the *Phapecoctavirus* (vB_EcoM_3A1_SA_NWU, vB_EcoM_10C3_SA_NWU, and vB_EcoM_118_SA_NWU) and *Vequintavirus* (vB_EcoM_366V_SA_NWU) had all LCBs arranged in the forward orientation while *Vequintavirus* phages (vB_EcoM_10C2_SA_NWU, and vB_EcoM_12A_SA_NWU) showed unique rearrangement of homologous modules, with eight LCBs arranged in the reverse complement orientation. Phage vB_EcoM_11B_SA_NWU (*Tequatrovirus*) revealed five LCBs arranged in the reverse complement orientation, with two of the local LCBs arranged in the forward orientation (Fig. 5). The whole genome sequence phylogenetic tree revealed all phages belonging to the same genera are closely related (Fig. 6). *Escherichia* coli O177 phages cladded together with closely related phages from the NCBI database, which indicated that these phages shared a common evolutionary history. TBLASTX analysis showed that *Phapecoctavirus*, *Tequatrovirus*, and *Vequintavirus* (vB_EcoM_10C2_SA_NWU and vB_EcoM_12A_SA_NWU) phages shared ≥ 96% identity similarity (with ˃ 95% coverage) and genome organisation (Fig. 7A–C). Phage vB_EcoM_366V_SA_NWU had low genome homology with other phages within *Vequintavirus* (Fig. 7B).

**Figure 8:**
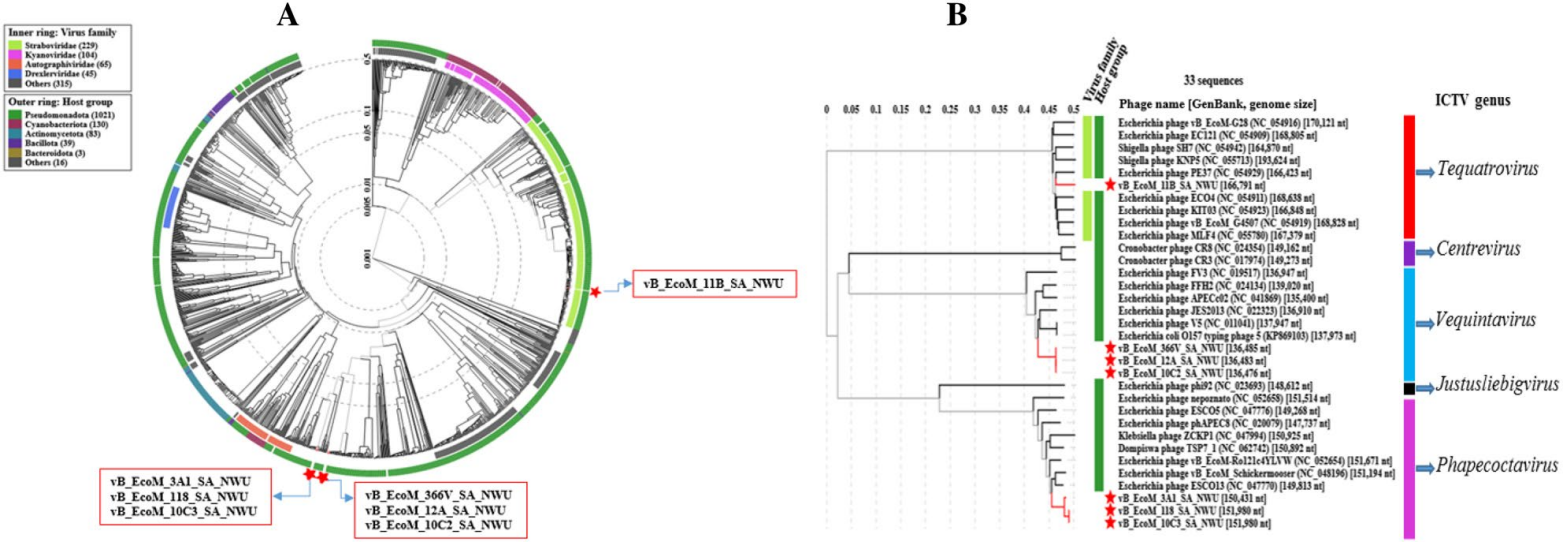
Viral phylogenetic tree (ViPTree) analysis based on genome-wide sequence similarities computed using tBLASTx. (**A**) ViPTree of 8 *E. coli* O177-infecting phage genomes and other 2480 phage genomes presented in the circular view. The coloured rings represent the virus families (inner ring) and host groups (outer ring) while the region marked with red asterisk represents the *E. coli* O177 phage genomes. (**B**) Rectangular phylogenetic tree (subset) of the phages generated using ViPTree, with the log scale on top representing the SG values. Red branches represent *E. coli* O177 phage genomes while black branches represent 26 linear dsDNA known phage genomes from ViPTree database. The right and left lines (green) represent the classification of the phages based on the host group and family level, respectively.

**Figure 9:**
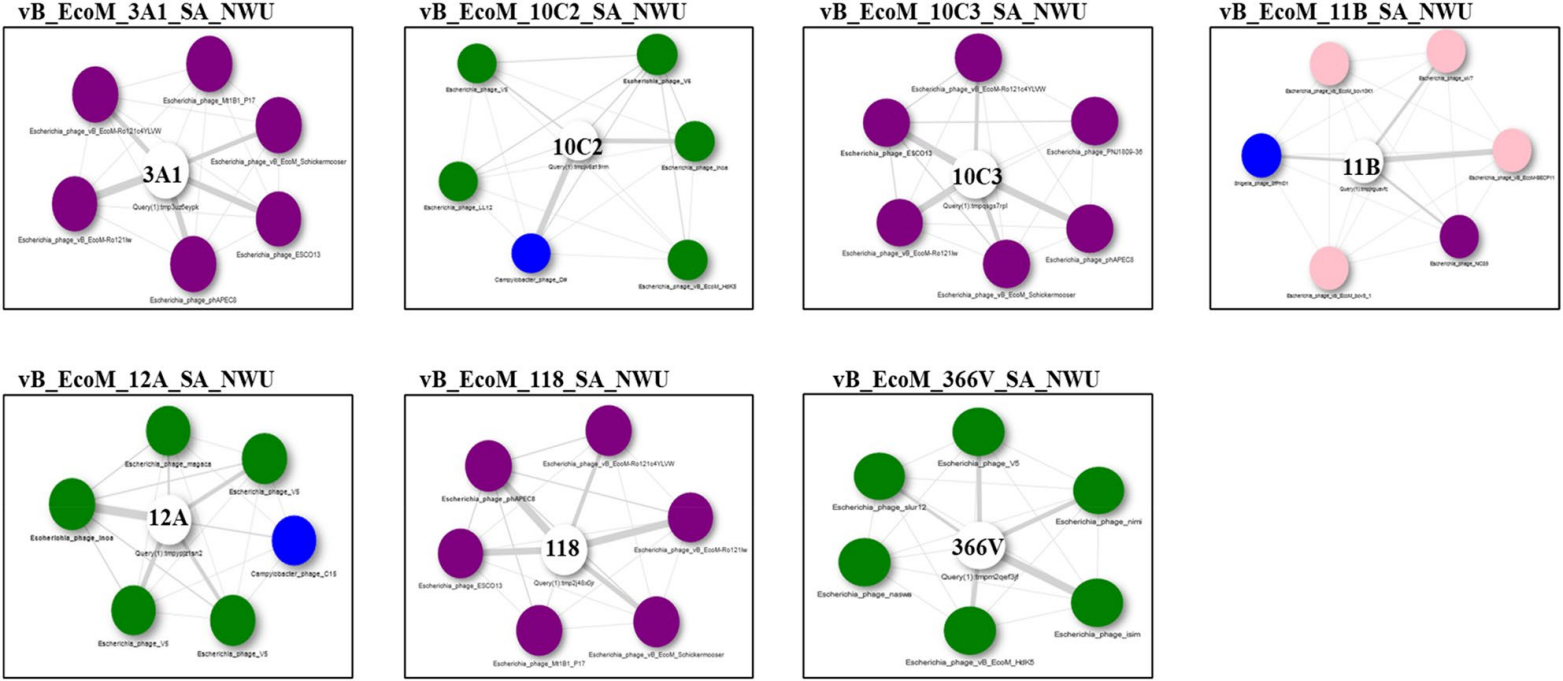
Phage cloud analysis of *E. coli* O 177 phage genomic relationship with the top six matched reference phage genomes from the NCBI-GenBank. Intergenomic distances computed by dashing based on a threshold of 0.15. The white node at the centre represents *E. coli* O177 phages.

**Figure 10:**
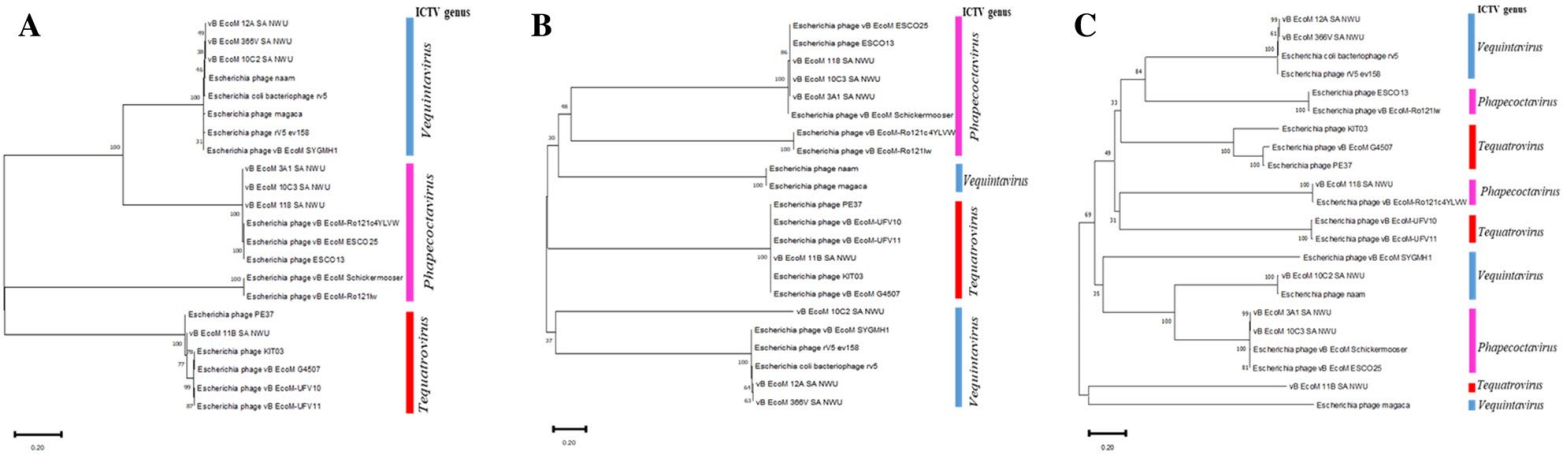
Phylogenetic neighbour-joining tree based on amino acid sequences of *E. coli* O177 phages and other *Escherichia* phages. (**A**) major capsid; (**B**) terminase large subunit (TerL), and (**C**) tail fiber protein. The trees were generated by MEGA 11 software using the neighbour-joining method and a bootstrap value of 1000 replicates.

ViPtree analysis showed high amino acid senteny among the *Phapecoctavirus, Tequatrovirus,* and *Vequintavirus* (Fig. 8A,B). *Escherichia coli* O177 phages clustered together with other phages (*Escherichia* phages) within the class *Caudoviricetes*, with the highest tBLASTx score (S_G_) level of > 0.99 (Fig. 8B). Phage vB_EcoM_11B_SA_NWU cladded separately from other *E. coli* O177 phages, which suggest a uniqueness at proteomic level. All the phages were classified into *Pseudomonadota* (synonym *Proteobacteria*) host group. Protein-to-protein network-based Phage cloud analysis linked *E coli* O177 phages with the phages belonging to the same genus with a distance range of 0.01–0.03 ratio (Fig. 9). The phylogenetic tree of the selected conserved protein sequences (major capsid, terminase large subunit (TerL), and tail fiber proteins) revealed various clustering patterns (Fig. 10A–C). As depicted in Fig. 10A, major capsid protein and TerL phylogenetic tree analysis showed that *E coli* O177 phages formed monophyletic clade together with their closely related *Escherichia* phages (from the representatives of *Phapecoctavirus, Tequatrovirus,* and *Vequintavirus* genera), which suggested that these proteins originated from a common ancestor. However, tail phylogenetic tree analysis generated different clustering patterns amongst the phages. Phages vB_EcoM_10C2_SA_NWU and vB_EcoM_11B_SA_ formed separate monophyletic group NWU (based on terminase large subunit and tail fiber protein sequences, respectively), which suggested a distinct relationship with other *Escherichia* phages from *Phapecoctavirus, Tequatrovirus* and *Vequintavirus*.

## Discussion

The emergence and transmission of AMR pathogens pose a significant threat to public health, especially considering limited novel antibiotic development^21^. Therefore, exploring alternative strategies like phage therapy is crucial. To develop phage therapy against AMR pathogens, it is essential to isolate and genomically characterise lytic phages. Accordingly, several studies have isolated and characterised phages that infect various pathogenic bacteria such as *A. baumannii*, *E. coli*, *K. pneumoniae*, *P. aeruginosa*, *Salmonella,* and *Vibrio* species^12,17,20,22–24^. Since lytic phage genome architecture harbours genes encoding several hypothetical proteins with unknown functions^22^, genomic analysis may help not only in the taxonomic classification of phages, but also identify good candidates for phage therapy.

The aim of this study was to characterise the complete genomes of seven *Escherichia* phages that infect multidrug resistant *E. coli* O177 strain isolated from cattle faeces. Genomic analysis showed that all phages contained linear dsDNA genomes spanning from 136,476 to 151,980 bp, with GC content that varied from 35.39 to 43.63%. Given that the genomes of *E. coli* O177 phages were smaller than 200 kbp, they were classified as small phages. These findings were consistent with previous studies, which have reported small phages infecting other pathogenic bacteria such as *E. coli*, *K. pneumoniae*, *P. aeruginosa*, and *Salmonella* species^10–14,25^. Although TEM analysis revealed that *E. coli* O177 phages had similar morphotype^20^, BLASTn and PhageAI analysis classified these phages under the subfamilies *Stephanstirmvirinae*, *Tevenvirinae*, and *Vequintavirinae* within the *Caudoviricetes* class. In addition, *E. coli* O177 phages were classified under three genera; *Phapecoctavirus* (vB_EcoM_3A1_SA_NWU, vB_EcoM_10C3_SA_NWU, and vB_EcoM_118_SA_NWU) *Tequatrovirus*, (vB_EcoM_11B_SA_NWU), and *Vequintavirus* (10C2_SA_NWU, vB_EcoM_12A_SA_NWU and vB_EcoM_366V_SA_NWU) as predicted by BLASTn and PhageAI tools. This suggests that phages presenting similar morphotype may belong to different families and/or genera. Interestingly, PhageAI results predicted all phages to be virulent, making them suitable candidates for phage therapy.

*Escherichia coli* O177 phages harboured a plethora of unique genes, which encode hypothetical and putative functional proteins. A substantial number of the CDSs and ORFs found in the phage genomes were predicted as hypothetical proteins with unknown functions. Similar observations have been reported in other phages that infect pathogenic bacteria species^10,22,26,27^. This indicates that phage genomes carry several genes whose functions are yet to be understood. Thus, research efforts must be directed at elucidating the true functions of these hypothetical proteins. Another interesting observation was that *E. coli* O177 phages contained genes coding for phage DNA replication, DNA synthesis and packaging, structural proteins (capsid and tail), and host lysis (lysozyme, and endolysin). In contrast with other phages, two phage genomes (vB_EcoM_3A1_SA_NWU, and vB_EcoM_366V_SA_NWU) contain genes encoding baseplate tail fiber, and tail spike proteins. Because tail fiber proteins are crucial for phage receptor recognition, a high abundance of baseplate, tail fiber, and tail spike proteins in a phage genome can enhance its infection capabilities and host range^28^. Phages also harboured tail tubular protein, whose enzymatic activity may hydrolyse polysaccharides and biofilm structures^29^. Indeed, tail tubular protein has been reported to play a role in bacteria cell lysis^30^, suggesting that *E. coli* O177 phages can also utilise tail tubular system to lyse the bacteria cell. Furthermore, *E. coli* O177 phage genomes carried the genes encoding DNA polymerase (DNAPs), RNA polymerase (RNAs), and RNA polymerase binding, which provide phages with a degree of autonomy when it comes to gene expression.

Genomes of the seven phages harboured genes encoding ribonucleoside-diphosphate reductase large subunit and ATP-dependent protease, which are involved in nucleotide and protein biosynthesis and metabolism. The ATPases have been reported to be a molecular motor, which catalyse the packaging process of the mature viral genome^31^. This makes the phages more self-sufficient with respect to their core replication machinery. Furthermore, the phages harboured genes coding for serine/threonine, activator middle promoter, Rha family transcriptional regulator, ADP-ribosyltransferase, and helix-turn-helix transcriptional regulator proteins. These proteins are involved in the transcription of viral early genes^10^. Moreover, our phage genomes harboured middle transcription regulatory protein motA and RNA polymerase proteins, which are responsible for activating middle and late prompters for the transcription of middle and later viral genes, respectively. It is worth noting that the ExPASY ProtParam analysis showed that lysozyme and endolysin proteins found in *E. coli* O177 phages are more stable and water soluble (GRAVY ranging between − 0.499 and − 0.261). The random helix predominated by α-helix contributes to the stability of secondary structures. Interestingly, in silico analysis showed that both proteins have a life span (in vivo) of ≥ 10 h in *E. coli* cell. Given that lysozyme and endolysin have antimicrobial activity, these attributes suggest that the proteins can be used as an antibacterial agent to combat AMR *E. coli* infections. Notably, no antibiotics resistance, virulence and/or lysogenic signatures were detected in any of the phage genomes.

While the exact roles of tRNAs in phages remain unclear, their presence in the phage genome is believed to enhance phage fitness and improve translational efficiency, potentially enabling independent translation from the host^5,17^. The tRNAs are frequently found in myoviruses with a large genome size^32^. Although tRNAs are commonly found in jumbo phages, some small/medium phage genomes may also carry tRNAs^10,17^. Similarly, tRNAscan-SE showed that our *E. coli* O177 phage genomes harboured 5–11 tRNA genes encoding various amino acids. Given that tRNAs play a key role in protein biosynthesis, these phages may have less tRNAs depended on the host translation mechanisms^17^. Interestingly, phages vB_EcoM_3A_SA_NWU and vB_EcoM_11B_SA_NWU possessed genes coding for other specialised tRNA enzymes (histidyl tRNA synthetase and valyl-tRNA synthetase), which may catalyse formation of tRNAs^10^. Histidyl tRNA synthetase, putative tRNA nucleotidyltransferase/poly(A) polymerase, and valyl-tRNA synthetase play a crucial role in protein synthesis by facilitating the attachment of amino acids to tRNA molecules^33^. Phage genomes also harboured genes coding for tRNA (Ile)-lysidine synthase that may play a role in tRNA maturation^34^. In addition, the tRNAs may contribute to high phage infectivity and virulence^8,35,36^.

Comparative genomic (VIRIDIC and ProgressiveMauve alignment) analysis revealed high sequence similarity between *E. coli* O177 phages and other *Escherichia* phages from *Phapecoctavirus*, *Tequatrovirus*, and *Vequintavirus* genera. These finding are consistent with the results obtained through BLASTn and PhageAI tools. Notably, VIRIDIC analysis showed that maximum intergenomic similarity scores ranged from 93.7 to 95.7% between *E. coli* O177 phages and other phages from the NCBI database. Based on 70% threshold, these findings suggest that *E. coli* O177 phages appear to be new members of *Phapecoctavirus*, *Tequatrovirus*, and *Vequintavirus* genera under *Stephanstirmvirinae, Straboviridae* (subfamily *Tevenvirinae*)*,* and *Vequintavirinae* families^4,37,38^. In addition, three phages (vB_EcoM_3A1_SA_NWU, vB_EcoM_10C3_SA_NWU, and vB_EcoM_118_SA_NWU) and *Escherichia* phage vB_EcoM-Ro1211w had intergenomic similarity of 95.7%, which suggests that these phages belong to the same species^37,38^. The Easyfig analysis also showed that genome organisation of *E. coli* O177 phages was similar to that of closely related phages. These finding support the results obtained by VIRIDIC analysis. Furthermore, ViPtree analysis revealed that *E. coli* O177 phages clustered with previously described phages from *Phapecoctavirus*, *Tequatrovirus*, and *Vequintavirus* genera, confirming that these phages share from a common ancestor. Similarly, phylogenetic tree analysis based on the conserved genes (major capsid, terminase large subunits, and tail fiber) supported the above findings.

## Conclusion

The current study provides insights into the genomic diversity of *E. coli* O177 phages. The results revealed that these phages harboured a vast array of hypothetical proteins with unknown function. In addition, these phages possessed genes encoding extra functions such as DNA replication, transcription, nucleotide metabolism, as well as lysis. The absence of antimicrobial resistance, lysogeny, and virulence signatures in *E. coli* O177 phages indicates that they are suitable candidates for biocontrol purpose. Genomic and proteomic analysis showed high similarity between *E. coli* O177 phages and other phages from the NCBI database. This suggests that our *E. coli* O177 phages are new members under the subfamilies *Stephanstirmvirinae*, *Tevenvirinae*, and *Vequintavirinae*.

## Materials and methods

### Propagation of phages

Seven bacteriophages, which were isolated from cattle faeces in our previous study^20^, were propagated using *E. coli* O177 strain. In brief, an aliquot of 100 µL of overnight culture of *E. coli* O177 was inoculated into 250 mL volumetric flasks containing 100 mL tryptic soya broth. A 200 µL aliquot of each phage lysate was added to the mixture. The flasks were incubated at 37 °C for 24 h in a shaking incubator (120 rpm). After incubation, the mixture was transferred into 50 mL falcon tubes and centrifuged at 10,000× g for 10 min. The supernatant was filter-sterilised using 0.22 µm pore-size acrodisc syringe filter. Phage stock was kept for the DNA extraction.

### DNA extraction and phage genome sequence

Phage genomic DNA was extracted using the phenol–chloroform protocol^39^, with minor modifications. Briefly, 1.5 mL of phage lysate was transferred into a 2 mL Eppendorf tube. The samples were treated with 18 µL of DNase (10 mg/mL) and 8 µL of RNAse A (10 mg/mL). The samples were mixed and incubated at 37 °C for 30 min. After incubation, the samples were treated with 50 µL of SDS (10%), 18 µL proteinase K (20 mg/mL), and 40 µL of 0.5 M EDTA (pH 8.0), followed by incubation at 60 °C for 60 min. Subsequently, 500 µL phenol–chloroform–isoamyl alcohol (25:24:1) was added to the samples. The samples were inverted five times and then centrifuged at 10,000× g for 5 min. The aqueous layer was transferred into a new 2 mL Eppendorf tube and mixed with 500 µL chloroform–isoamyl alcohol (24:1), inverted five times, and centrifuged at 10,000× g for 5 min. The aqueous layer was transferred into a new 1.5 mL Eppendorf tube and mixed with 45 µL of 3 M sodium acetate (pH 7.5) and 500 µL isopropanol (100%). The samples were incubated at − 20 °C overnight. Subsequently, the samples were centrifuged at 14,800 × g for 30 min. The DNA pellet was washed with three times pre-chilled 70% alcohol. The DNA pellet was air-dried, resuspended in 70 µL of 1X TE buffer (10 mM Tris PH 8.0, 1 mM EDTA), and stored at − 20 °C.

Prior to genome sequencing, the DNA samples were purified using GeneJet PCR Purification Kit (ThermoFishre, Baltics UAB, Lithuania) following the protocol described by the manufacturer. Subsequently, the samples were transported to Inqaba Biotechnical Industries (Pty) Ltd for sequencing. The DNA libraries were prepared using the NEBNext^®^ Ultra™ II DNA Library Preparation Kit for Illumina^®^ (NEB, Ipswich, MA, USA) according to the manufacturer’s instructions. The libraries were quantified using Qubit 2.0 fluorometer (Thermo Fisher Scientific Inc., Waltham, MA, USA) and genome sequencing was performed using Illumina NextSeq sequencing platform. A total of 400 Mb data (2 × 150 bp paired-end reads) was generated per sample.

### Bioinformatics analysis and phage genome annotation

The quality of the raw reads was assessed using FastQC (v0.11.9)^40^, and then the adapters, N bases, and low-quality reads were removed using Trimmomatic (v0.36)^41^. The reads with high quality score were assembled into contigs using Spades v3.15.3, with K-mer set at default^42^. The assembled contigs were annotated using the Rapid Annotation using System Technology Toolkit (RASTtk) pipeline^43^. The annotated proteins were manually curated and validated using UniProtKB (https://www.uniprot.org/, accessed on 20 February 2023). The complete genomes were compared with other phage genome sequences using BLASTn from the NCBI database (https://blast.ncbi.nlm.nih.gov/Blast.cgi, accessed on 20 February 2023). Phage taxonomy and lifestyle were computationally predicted using BLASTn (https://blast.ncbi.nlm.nih.gov/Blast.cgi) and PhageAI tools^44^. Potential open reading frames (ORFs) were predicted and annotated RAST (http://rast.nmpdr.org, accessed on 23 February 2023), GeneMark (http://opal.biology.gatech.edu/GeneMark/, accessed on 23 February 2023) and NCBI ORFfinder tool (https://www.ncbi.nlm.nih.gov/orffinder/, accessed on 23 February 2023). Predicted ORFs were manually validated and curated. Subsequently, homology searches for each identified ORF sequences were subjected to BLASTp against the non-redundant protein database (with parameters set at score of > 50, E-value of < 1.0 × 10^−2^)^45^. Codon usage frequencies in the phage genomes were computed using Codon Usage programme (https://www.bioinformatics.org/sms2/codon_usage.html, accessed on 4 March 2023). The sequences were used to search for the protein domains in InterProScan 5 (http://www.ebi.ac.uk/interpro/search/sequence/, accessed on 4 march 2023)^46^, and protein family (Pfam) database (http://pfam.xfam.org/, accessed on 4 March 2023)^47^.

The presence of tRNAs were predicted using ARAGORN and tRNAscan-SE tools^48,49^. The rho–independent transcription terminators were determined using ARNold (http://rssf.i2bc.paris-saclay.fr/toolbox/arnold/index.php, accessed on 10 March 2023) and Genome2D tools^50^. Putative promoters were searched using the phage promoter integrated in Galaxy platform v 0.1.0 (https://bit.ly/2Dfebfv, accessed on 10 March 2023) with the parameters set at: thresholds: 90%, phage family: *Myoviridae* (former), host bacteria genus: *Escherichia coli*, and phage type: virulent^51^. Possible anti-CRISPR genes were predicted using AcrDB tool (https://bcb.unl.edu/AcrFinder/, accessed on 4 March 2023)^52^. The presence of virulence determinants was screened using VICTORS^53^, and Virulence Factor Database (VFDB)^54^, while antimicrobial resistance, and temperate genetic signatures were determined using Comprehensive Antibiotic Research Database (CARD)^55^, and PhageLeads^56^.

### Domain identification and prediction of *E. coli* O177 phages structural proteins by homology modelling

The domains on proteins were ascertained using HmmerWeb (v2.41.2)^57^, and the protein family database^47^. Topology of proteins was analysed for the presence of transmembrane and signal-arrest-release (SAR) domains as previously described^58–60^. Signal peptides were predicted using Phobius (v. 1.01)^61^, Topcons (v.1.0)^62^, SOSUI (v. 1.1)^63^, DeepTMHMM (v1.0.12) and SignalP-6.0 (v0.0.52)^60^. Physiochemical properties and secondary structure of lysozyme and endolysin proteins were predicted using the ExPASY ProtParam tool (https://web.expasy.org/protparam/, accessed on 12 April 2023) and SOPMA (https://npsa-prabi.ibcp.fr/cgi-bin/npsa_automat.pl?page=/NPSA/npsa_sopma.html, accessed on 12 April 2023), respectively. Homology modelling was performed on lysozyme and endolysin proteins found in *E. coli* O177 phage genomes. Each protein sequence was used as a query to identify templates using HHPred server (https://toolkit.tuebingen.mpg.de/tools/hhpred, accessed on 20 April 2023)^64^. The templates with highest identity similarity of ≥ 95%, and E-value of ≤ 0 for each sequence (top hits) were selected to generate structural models using web-based MODELLER (v10.0) algorithm^64,65^. The predicted 3D structures were verified for accuracy by VERIFY_3D (https://saves.mbi.ucla.edu/, accessed on 20 April 2023), PROSA (https://prosa.services.came.sbg.ac.at/prosa.php, accessed on 20 April 2023), and PROCHECK (http://www.ebi.ac.uk/thornton-srv/software/PROCHECK/, accessed on 20 April 2023).

### Comparative genomics and proteomics analysis of phages

The intergenomic nucleotide sequence similarity among *E. coli* O177 phages and other *Escherichia* phages from the GenBank database was determined using Virus Intergenomic Distance Calculator (VIRIDIC). The genomic similarity threshold was set at 70% for genus and 95% for species^37^. Whole genome comparison between *E. coli* O177 phages and closely related *Escherichia* phages was performed using progressiveMauve software (v2.3.1)^66^, and tBLASTx on the DiGAlign Dynamic Genomic Alignment sever (https://www.genome.jp/digalign, accessed on 10 June 2023)^67^. Linear genome map comparison of the phages belonging to the same genus were created using Easyfig (v2.2.5) (http://mjsull.github.io/Easyfig/, accessed on 20 June 2023), using tBLASTx^68^.

Viral proteomic tree (ViPTree) server (v3.1) (https://www.genome.jp/viptree/, accessed on 20 June 2023) was used to infer a tree of *E. coli* O177 phages and other known phage genome sequences based on genome-wide sequence similarities computed using tBLASTx^67^. In addition, Phage clouds analysis was used to visualise the genomic relationship between *E. coli* O177 phages and other phages in NCBI database based on their genomic distances (with the threshold of 0.2)^69^. The amino acid sequences of three conserved proteins sequences (major capsid, terminase large subunit (TerL), and tail fiber) were used to construct phylogenetic tree by the neighbour-joining method using MEGA 11 with 1000 bootstrap repeats^70^. The complete genome sequences were deposited into the NCBI database, and the accession numbers are stated in Table 1.

### Supplementary Information

Supplementary Table S1.

Supplementary Table S2.

Supplementary Table S3.

Supplementary Figures.

## Data Availability

Sequence data generated and presented in this study have been deposited into the NCBI database under the GenBank Accession numbers; https://www.ncbi.nlm.nih.gov/nuccore/OR062524; https://www.ncbi.nlm.nih.gov/nuccore/OR062525; https://www.ncbi.nlm.nih.gov/nuccore/OR062526; https://www.ncbi.nlm.nih.gov/nuccore/OR062527; https://www.ncbi.nlm.nih.gov/nuccore/OR062528; https://www.ncbi.nlm.nih.gov/nuccore/OR062529; https://www.ncbi.nlm.nih.gov/nuccore/OR062530.
