# Supplementary material for: Comparative genomics and proteomics analysis of phages infecting multi-drug resistant Escherichia coli O177 isolated from cattle faeces
Source: Sci Rep. 2023 Dec 5;13:21426. doi: 10.1038/s41598-023-48788-w (PMC10698182; doi:10.1038/s41598-023-48788-w)
Supplement: Supplementary file 4 — Supplementary Figures. [file 41598_2023_48788_MOESM4_ESM.pdf]

Comparative genomics and proteomics analysis of phages infecting multi-drug resistant *Escherichia coli* O177 isolated from cattle faeces

Peter Kotsoana Montso[1,2,*], Andrew M. Kropinski[3], Fortunate Mokoena[4], Rian Ewald Pierneef[5,6,7], Victor Mlambo[8], Collins Njie Ateba[1,2]

[1]Food Security and Safety Focus Area, Faculty of Natural and Agricultural Sciences, North-West University, Private Bag X2046, Mmabatho 2735, South Africa.

[2]Department of Microbiology, Faculty of Natural and Agricultural Sciences, North-West University, Private Bag X2046, Mmabatho 2735, South Africa.

[3]Department Food Science, and Pathobiology, Ontario Veterinary College, University of Guelph, Guelph, ON N1G 2W1, Canada

[4]Department of Biochemistry, Faculty of Natural and Agricultural Sciences, North-West University, Mmabatho, South Africa.

[5]Department of Biochemistry, Genetics and Microbiology, University of Pretoria, Pretoria, 0001, South Africa

[6]Centre for Bioinformatics and Computational Biology, University of Pretoria, Pretoria, 0001, South Africa

[7]SARChI Chair: Marine Microbiomics, microbiome@UP, Department of Biochemistry, Genetics and Microbiology, University of Pretoria (UP), Hatfield, Pretoria, South Africa

[8]School of Agricultural Sciences, Faculty of Agriculture and Natural Sciences, University of Mpumalanga, Mbombela, 1200, South Africa.

[*]Correspondence: montsokp@gmail.com

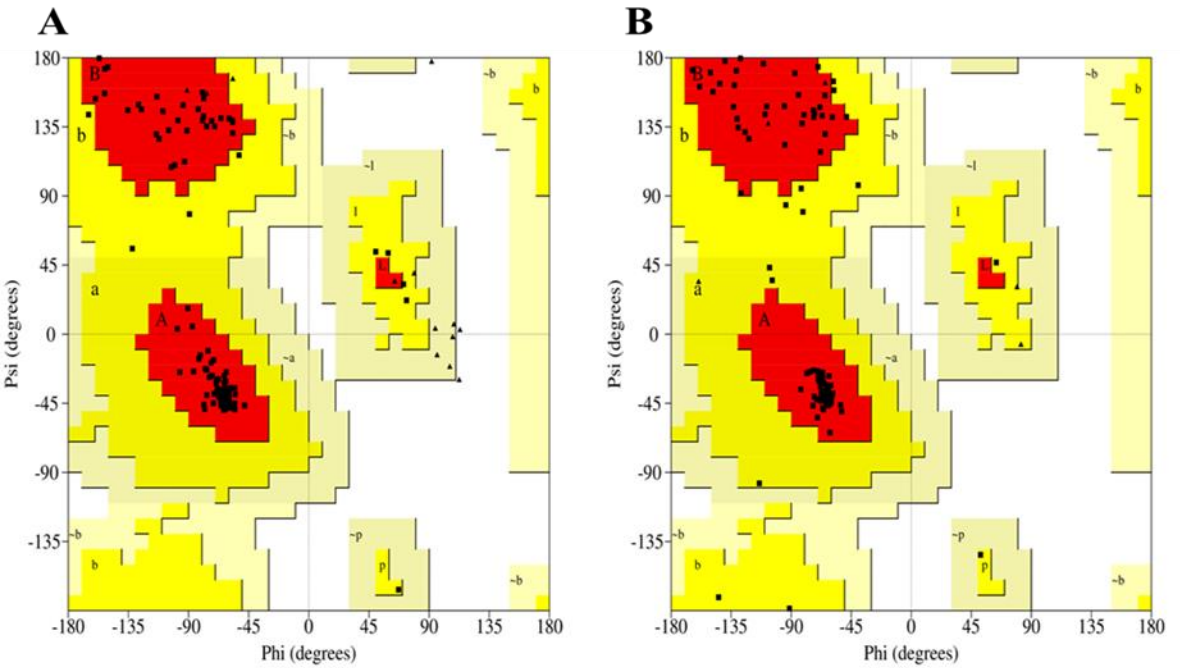

Supplementary Figure 1: Ramachandran plot depicting the phi-psi torsion angles for all the residues of the models (**A** = lysozyme, and **B** = endolysin proteins) predicted by PROCHECK. Red colour represents the most favoured region while yellow colour represents the allowed region, and white colour represents disallowed region. The A, B, L: Most favoured regions; a, b, l: additional allowed regions; ~a, ~b, ~l, ~p: generously allowed regions.

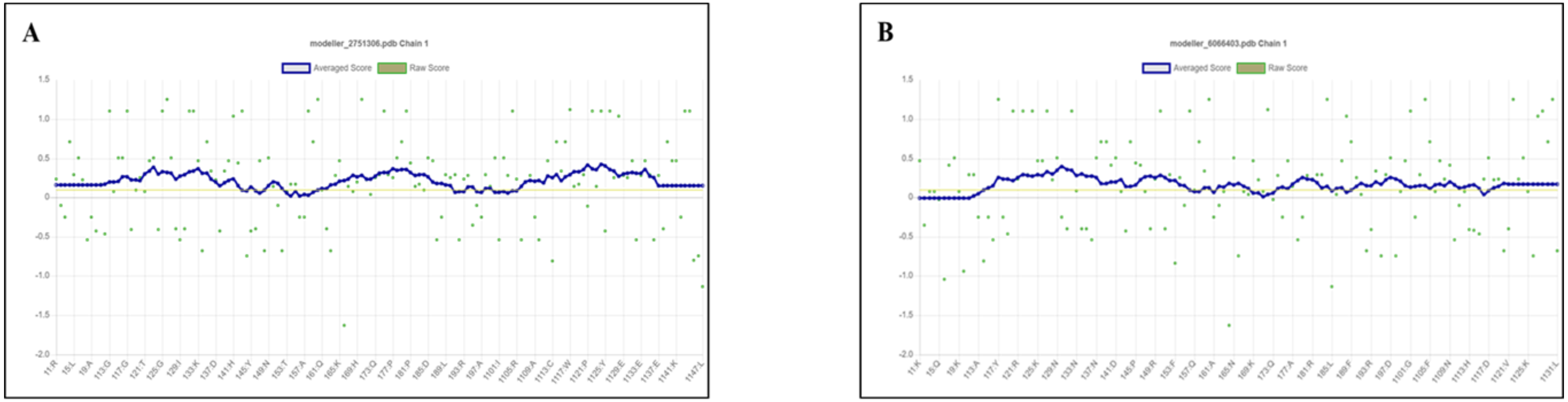

Supplementary Figure 2: The VERIFY_3D plot showing the models (**A** = lysozyme, and **B** = endolysin proteins). The blue line represents the average score, which is the standard to judge the quality of the structure; it is considered as a high-quality model when more than 80 % of the residues have 3D-D score of higher than 0.2.

The VERIFY_3D plot for.

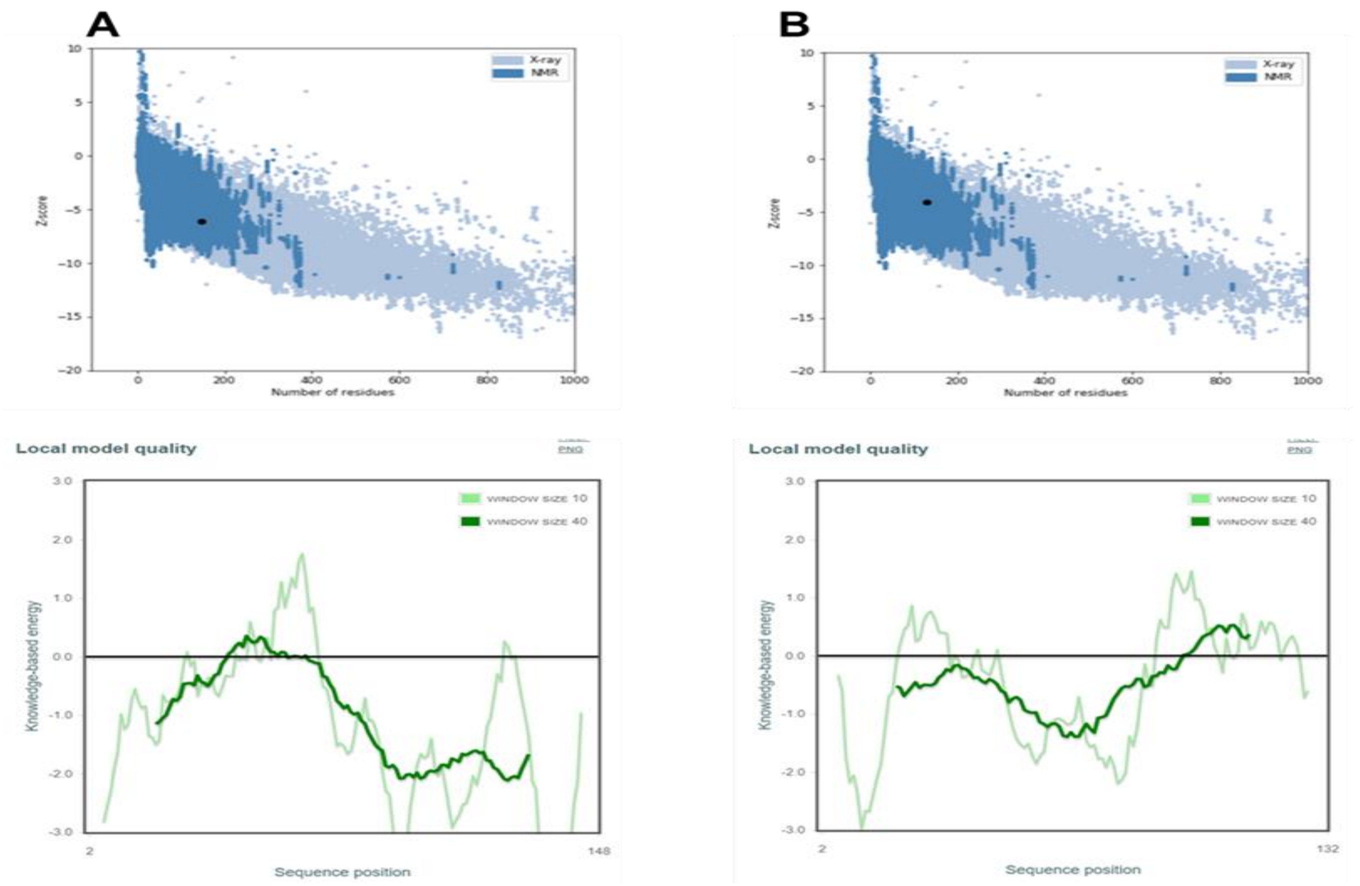

Supplementary Figure 3: ProSA sever Z-score of the final models (**A** = lysozyme, **B** = endolysin and, **C**= tail fiber proteins). The Z-score plots (Upper part) shows the Z-score (black dots). The energy plot (lower part) as computed by PROSA server.
